# Male and Female C57BL/6 Mice Respond Differently to Awake Magnetic Resonance Imaging Habituation

**DOI:** 10.3389/fnins.2022.853527

**Published:** 2022-06-10

**Authors:** Thomas Beck Lindhardt, Eugenio Gutiérrez-Jiménez, Zhifeng Liang, Brian Hansen

**Affiliations:** ^1^Department of Clinical Medicine, Center of Functionally Integrative Neuroscience, Aarhus University, Aarhus, Denmark; ^2^CAS Center for Excellence in Brain Sciences and Intelligence Technology, Institute of Neuroscience, Chinese Academy of Sciences, Shanghai, China

**Keywords:** awake, MRI, sex, stress, rodent, habituation

## Abstract

Traditionally, preclinical magnetic resonance imaging (MRI) has been performed in anesthetized animals. However, anesthesia has been shown to perturb normal brain function and physiology. Such effects limit our ability to detect subtle physiological alterations in disease models and treatment studies, thus hampering discovery and compromising generality of findings. Therefore, methods for awake animal MRI are needed to study the rodent brain in its natural physiological state, free of anesthetics. Current setups for awake animal MRI rely on restraining systems to avoid animal movement during scanning. To reduce restraint stress, animals are habituated to the scanner environment prior to MRI data collection. To date, however, most awake MRI studies employ male rodents only. This is a fundamental limitation as results obtained may be pertinent only to half of the population. We characterized training and habituation responses of male and female mice to provide improved, sex-dependent training procedures for awake mouse MRI. We recorded heart rate, monitored behavioral responses (body weight and *fecal boli* weight) and fecal corticosterone levels (FCM) as indicators of wellbeing and stress during a 14-day progressive habituation protocol. In addition, we also assessed discomfort levels and anxiety using the mouse grimace scale (MGS) and light/dark test (LDT), respectively. All scores were compared between both groups. We found that heart rate was significantly decreased after 10 and 11 days of training for both males and females, respectively. However, the specific time course for this decrease was significantly different between males and females, and females exhibited higher anxiety levels during habituation and 14 days after habituation than males. Lastly, we also found that mean FCM levels for both groups were decreased after 11 days of MRI habituation. The present work shows that mice can be successfully trained for extended MRI sessions which is necessary for many (particularly non-fMRI) studies. Importantly, we find that males and females differ in their response to awake MRI habituation, which should be considered in future awake MRI studies that aim to include male and female mice.

## Introduction

Anesthetics have long been standard practice and a necessity in basic and preclinical neuroscience using rodent magnetic resonance imaging (MRI) (Ferris et al., 2011). During scanning, even minor head motion by the animal can distort the acquired images, thus corrupting the data set or at best complicating the data analysis and subsequent interpretation of the results (Hajnal et al., 1994). For this reason, anesthetics have been used to provide animal immobility while simultaneously ensuring signal acquisition with low stress and high reproducibility (Hanusch et al., 2007). Recently, other strategies based on trained, awake animals have emerged (King et al., 2005; Yoshida et al., 2016; Han et al., 2019). This effort is partly driven by the well-known fact that anesthesia modifies various physiological parameters and processes such as vascular reactivity, brain metabolism, and neuronal and glial activities (Gao et al., 2017). Despite these shortcomings, anesthesia application in rodent MRI remains a standard, and much effort has also gone into optimizing anesthetic protocols to minimize physiological alterations caused by different anesthetic agents (Schlegel et al., 2015; Sharp et al., 2015; Petrinovic et al., 2016; Shim et al., 2018). However, many types of experiments such as task-based functional (f) MRI or comparisons of the awake with the sleep state cannot be performed in the anesthetized animal. Therefore, the transition to awake imaging is necessary to refine rodent MRI as a translational tool. Thus, in recent years an array of new MRI *in vivo* methods have emerged for imaging both rats and mice in the awake state (Ferris et al., 2011). The main advantage of transitioning to awake MRI is the ability to acquire imaging data that remain completely unperturbed by the confounding effects of anesthesia, thus improving the study of the brain in its natural working state (Peeters et al., 2001; Wang et al., 2010). Another consideration is the widespread use of awake animals in optical imaging of the brain. This is somewhat easier to implement as these microscopy methods are largely silent in comparison to MRI and therefore less stressful for the animal. In order for such optical techniques to fully supplement preclinical MRI, the transition to awake MRI is needed as data are otherwise acquired in vastly different physiological states. As of today, awake MRI in rodents has already been utilized in a broad variety of neuroscience applications, including awake fMRI of the default mode network (Yoshida et al., 2016; Madularu et al., 2017) alone or combination with optogenetic stimulation (Desai et al., 2011), chemogenetics (Giorgi et al., 2017), sensory stimulation (Harris et al., 2015; Chen et al., 2020), behavioral tasks (Han et al., 2019), assessment of cortical plasticity (Matsubayashi et al., 2018), drug response (Moore et al., 2016), glymphatic function (Stanton et al., 2021), and various disease models (Ferris et al., 2014). Some MRI studies, however, require extensive scan protocols either due to the need to acquire multiple data sets for analysis (e.g., for quantitative MRI combining diffusion, perfusion, and relaxometry) or gated acquisitions where final scan time depends on the animal respiration pattern. For such studies to be feasible with awake animals a validated, extended training scheme as the one presented here is needed.

When conducting awake rodent MRI, other key technical and methodological challenges arise. One major challenge is to minimize the awake animal’s body and head motion levels during the live imaging sessions. In previous awake rodent MRI studies, minor and stable body and head motion levels have been accomplished by either surgically implanted head holders or by (Bergmann et al., 2016; Chang et al., 2016; Yoshida et al., 2016; Han et al., 2019; Chen et al., 2020) using less invasive restraining kits that utilize pads securing the head and lower body (abdomen) (Madularu et al., 2017) to restrain the animals. Without habituation, however, restraining can induce severe stress that in turn might confound the acquired imaging data (Asaad and Lee, 2018). Therefore, animals need to undergo repeated habituation sessions to be comfortable not only with being restrained but also with the loud acoustic noise caused by the MR gradients during scans. The rodent habituation can either be conducted using an available MRI scanner, a mock MRI environment, or a mix of both (Chen et al., 2020).

To ensure that animals are sufficiently habituated to the MRI environment, many different physiological and behavioral parameters can be used as proxy measurements of animal’s stress levels. These parameters include heart rate (Ferris et al., 2011), respiratory rate (Ferris et al., 2011; Chang et al., 2016; Madularu et al., 2017), body weight (Yoshida et al., 2016), amount and weight of *fecal boli* (Yoshida et al., 2016; Madularu et al., 2017), corticosterone blood plasma levels (Ferris et al., 2011), ultrasonic calls, and motion-associated fMRI variance (King et al., 2005; Madularu et al., 2017). Monitoring these parameters over the habituation period is essential because it is still not fully understood how rodents are affected (short-term and long-term) by the currently employed habituation procedures. This lack of insight holds back refinements to the habituation procedures and clouds potential differences that male and female rodents may exhibit. Limitations aside, continuous MRI habituation is reported by several groups to decrease rodent stress overall and reduce stress-related behavior. However, no attention has been devoted to investigating if sex differences in habituation response exist for neither rats nor mice. For long, many researchers have relied exclusively on male rodents for animal experiments, primarily to avoid the physiological variability linked with the estrous cycle of female rodents. However, focusing solely on males severely holds back research, as biological results, from a translational perspective, may only be relevant to half of the population (Mauvais-Jarvis et al., 2017). With the steady increase in attention to the use of female subjects in science (Luine et al., 2017) and the rise in popularity of awake preclinical MRI (Ferris et al., 2011), it is crucial to elucidate sexual differences in response to habituation. It is well-acknowledged that male and female rodents respond differently to stressors (Luine et al., 2017; Rincón-Cortés et al., 2019) and exhibit different anxiety-related behaviors (An et al., 2011). For example, female rodents secrete a higher concentration of CORT in response to physical and physiological stressors than do their male counterparts. In addition, female rodents also exhibit greater concentrations of corticosterone at baseline (Rincón-Cortés et al., 2019). Therefore, it is likely that male and female rodents will differ in their responses during habituation procedures. If true, such findings would require researchers to take these differences into account when habituating mice for awake MRI.

For these reasons, this study aimed to investigate the potential sex differences that mice exhibit in response to repeated MRI habituation. For this, we developed our setup for awake mouse MRI habituation relying on surgical implants previously established (Han et al., 2019; Chen et al., 2020) for head fixation. We monitored heart rate, body weight fluctuations, *fecal boli* weight and assessed corticosterone concentration levels during the length of the MRI habituation protocol and compared data between the sexes. Corticosterone measurements were also conducted prior to animal handling and 2 weeks after the MRI habituation had ended. The level of the animal’s discomfort during MRI habituation session were also assessed by scoring images from the facial video feed using the mouse grimace scale (MGS). In addition, we employed the light/dark anxiety test (LDT) to assess mouse anxiety-related and locomotor activity responses to awake MRI habituation procedure and compared these parameters between males and females. Finally, we repeated the LDT 14 days after the end of the MRI habituation protocol. In general, male and female mice respond to MRI habituation in ways consistent with previous awake mouse MRI studies aiming for habituation for shorter scan sessions. Habituation for extended awake MRI scan sessions is therefore possible. However, male and female mice show distinctly different responses through the course of our training protocol meaning that 14-day habituation is needed in order for both sexes to be ready for awake MRI.

## Materials and Methods

### Animals

Naïve male (*n* = 20) and female (*n* = 22) 6-week-old *C57BL/6* (Taconic Bioscience Inc., Ejby, Denmark) mice were used during this study. A subset of male (*n* = 9) and female (*n* = 9) mice were chosen for fecal corticosterone metabolite assessment and were not used for physiological recordings of heart rate. Three of those males and three of those females were used in control experiments and did not undergo surgery for head-holder implantation nor received MRI training. Upon arrival at our facility, mice were given 2 weeks of acclimatization to the stable environment before proceeding with the experimental protocols. Mice were housed sex-wise in cages of up to three on a 12-h dark/light cycle (5:00 a.m. to 5:00 p.m.) with *ad libitum* access to food and water.

Animals were housed with a minimum of one cage-mate as single-housing of animals has been shown to disrupt sleep patterns and increase resting-state heart rate as well as increase blood plasma corticosterone levels (Späni et al., 2003; Olsson and Westlund, 2007). Temperature and humidity were controlled at 21°C ± 2 and 45% ± 5, respectively. All cages were supplied with bedding material and cage enrichment (nesting material, wooden chew sticks, and two different hideouts). All mice were handled by a single experimenter throughout the experiment to minimize stress induced by multiple handlers. Body weight was monitored post-surgery and throughout the whole experimental period. Body weight loss exceeding 20% of normal bodyweight was considered a humane endpoint.

**FIGURE 1:**
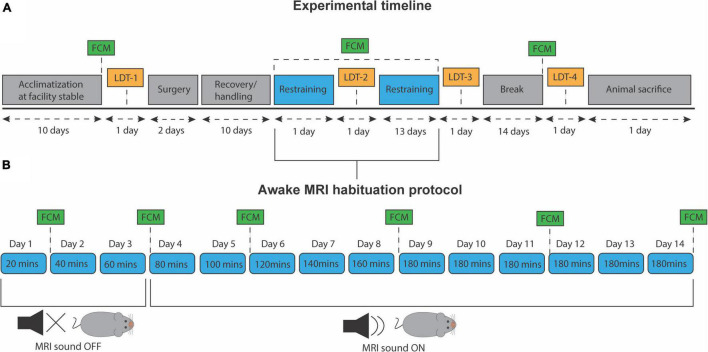
Experiment timeline and awake MRI habituation protocol. **(A)** Timeline for all experimental procedures. LDT, light/dark test. FCM, fecal corticosterone metabolite assessment. **(B)** Progressive awake MRI habituation protocol. Twenty minutes were added to each session per day, up to a total of 180 min. MRI sound was played from day 4 and onward.

After the experimental period, all animals were euthanized with barbiturate (Pentobarbital, 1,300 mg/kg) with a maximum injection volume of 0.5 mL. All animal housing, handling, and experimental protocols were conducted according to the regulations of the Danish Ministry of Justice and Animal Protection Committees under permit no. (2019-15-0201-00285). The experiment timeline and habituation protocol are outlined in Figures 1A,B, respectively.

### Mock Magnetic Resonance Imaging Environment and Head-Holder

The animal bed for awake application and the head-holder used for fixation are custom-made 3D models, adapted from Han et al. (2019) to fit our current setup^1^ using a rat-cryoprobe. The 3D models of the animal bed, head-holder, and all components for the mock MRI environment were modified and designed using Autodesk Fusion 360 software. A library of our designs is available on our GitHub repository: https://github.com/CFIN-High-Field-MRI

**FIGURE 2:**
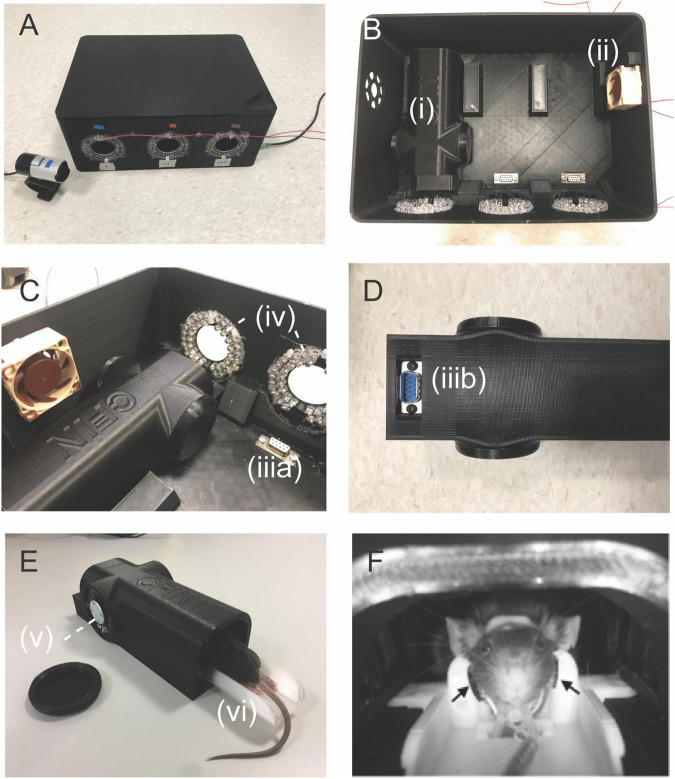
Awake MRI setup and components. **(A)** Awake MRI habituation box where animals are trained during the awake MRI habituation sessions. **(B)** Overview of the inside of the habituation box with connections for three chambers (i) with one animal inside each. A small fan provided ventilation of the habituation box (ii) positioned at the side of the box. **(C)** Sideview of inside the habituation box. The chambers were connected to the audio sockets (**C**,iii) positioned at the bottom of the box. IR diodes provided illumination of the animals for video recordings. **(D)** Underneath the chamber. Each mock coil had an audio connector **(D**,iii) connected to (**C**,iii). **(E)** Sideview of the mock MRI coil. The animal bed (iv) was inserted into the chamber (i), and each chamber had speakers installed (v) at the left and right ear of the mouse. **(F)** Webcam recording of the restrained animal on the MRI bed inside the habituation box. Black arrows: placement of the light sensor (carotid arteries).

The simulation environment consists of a habituation box (Figure 2A) closed with a lid during habituation, as the scanner bore is also dark during scans. The habituation box can accommodate three chambers (Figure 2Bi) which emulate the inside of the scanner bore with a cryo-probe where we perform the MRI scans. Inside the habituation box, a mini-fan is installed for ventilation (Figure 2Bii). Three audio sockets (Figure 2Ciii) are installed on the bottom of the habituation box to fit each chamber’s audio connector (Figure 2Diii). Three viewports allow for webcam insertion, and infrared (IR) light-transmitting diodes (Figure 2Civ) are installed around each viewport to illuminate the rostral region of the mouse. The IR diodes are connected in series and powered by a nearby power supply (Elektro-Automatik, Art.Nr: 03100206). The chambers are equipped with speakers (Figure 2Ev) on either side of the animal’s head to play scanner sounds during habituation. The MRI audio sequences are played using Audacity software (Audacity Team), and audio is transmitted *via* an auxiliary cable that connects to the audio sockets of the habituation box. During each habituation session, the experimental MRI sound was played at 110 dB. The chambers for one animal bed and animal (Figure 2Evi). Webcam recordings allowed us to observe the animals while restrained inside the habituation box (Figure 2F). Our habituation environment captures salient features of the scan situation including identical beds, light conditions, and sounds. Vibrations are not a concern in our scanner setup as our cryo-probe is suspended in the scanner bore so there is no contact between the gradients and the animal bed/receiver coil. Gradient vibrations are therefore not transferred to the animal bed. For this reason, our simulation chamber does not include this effect.

### Head-Holder Implantation

All surgeries conducted in this study were based on Han et al. (2019). Mice were placed in an airtight box and briefly anesthetized with isoflurane (4%). The top of the skull was then carefully shaved, and the mice were placed onto a stereotaxic frame (RWD Life Science). Anesthesia was maintained with a mixture of 1.2–1.5% isoflurane, 0.6 mg/l medical air and 0.4 mg/l oxygen. The shaven area was then sterilized with ethanol, and a drop-shaped piece of skin was removed from the head to uncover the skull. Aponeurosis and periosteum were carefully removed alongside any muscle tissue on the interparietal bone posterior to lambda. The exposed skull was cleaned with saline, dried out with ethanol, and then the tissue edges were fixed to the skull using a layer of tissue adhesive (3M, Vetbond). The uncovered part of the skull was then scraped with a surgical knife to produce a hatch pattern (gridlines) to enlarge the surface area for better adhesion. Next, a thin layer of light-curing self-etch (3M, ESPE Single Bond Universal Adhesive) was applied to the midline and on the interparietal bone area and subsequentially cured with blue light. Then, a layer of light-curing flowable dental resin (Ionoseal, Voco, Germany) was applied on the interparietal bone before placing the custom head holder. A 1 mm in diameter alumina ceramic rod (KF Advanced Ceramic, China) was then placed down the midline and in-between the gap of the head holder to strengthen the build. Lastly, the rod and holder were secured by curing the resin and more light-curing flowable resin was applied around the holder. The remainder of the exposed skull was covered with a thin layer of dental cement, and the mouse was tagged for easy identification. After surgery, all mice received a daily dose of antibiotics; 0.2 mg/g (STADA, Ampicillin, Vnr: 108259), anti-inflammatory drugs; 0.1 mg/g (ScanVet, Carprofen, Vnr: 027693) and painkillers; 0.12 mg/g (Indivior, Temgesic, Vnr: 521634) for a minimum of 4 days. Ampilicin and Carprofen were administered IP while Temgesic was administered SC post-surgery and diluted (2 ml Temgesic/120 ml water) in their drinking water for the remainder. In total, mice were given 10 days of recovery.

### Habituation Protocol

During the last 3 days of the recovery period, all mice received 20 min of daily handling. This was done to reduce stress levels and ensure that animals were familiar with the caretaker before the awake MRI habituation. A copy of the 3D-printed, experimental animal bed was also placed in their cage at all times to let them familiarize themselves with the component. After each session, mice were given a few droplets of condensed milk as a reward. On day one of habituation, mice were carefully introduced to the setup and restrained for 20 min in total. Twenty minutes were added in the following training session until reaching a total of 180 min of habituation (Figure 1B). Only on the fourth day and onward, the MRI sounds were played. Before each awake MRI habituation session that included the MRI sound, animals were given wax earplugs (Ohropax Classic). Animals were trained sex-wise, nine per day but were divided into three different timeslots; morning (7:30–10:30), noon (11:00–14:00), and afternoon (14:30–17:30). During the entire habituation period, the male and female mice groups were staggered to be equally divided into the three different timeslots. In total, animals received 14 days of habituation (Figure 1). The animal experiments were conducted in mixed-sex batches of nine animals to ensure data acquisition homogeneity across animal sex and minimize experience bias. However, to minimize the influence of different odors on mice behavior, animals’ sex was not mixed during the habituation sessions.

#### Video Monitoring During Awake Mice Habituation Protocol

Mouse body movement was monitored for each habituation session using webcams (LifeCam Studio, Microsoft) modified to allow for infrared light detection. The webcams were positioned through the viewports of the box (Figure 2A), so each animal was visible (Figure 2F). The video files were stored to assess animal discomfort during habituation using the mouse grimace scale (MGS). We used three frames; early (first minute), mid (±1 min), and late (last minute), for assessment of the MGS. All images were rated on the MGS while randomized and the experimenter blinded. The data was preprocessed in MATLAB (The Mathworks Inc., R0219a), and statistical analysis was done using Prism 9 software (GraphPad).

#### Peripheral Physiological Recordings and *Fecal boli* Collection During Awake Mice Habituation Protocol

Heart rate and respiratory rate were collected during each habituation session using pulse oximetry (MouseOx, STARR Life Science, Oakmont, PA). The clamp of the light-sensor device (MouseOx, “Throat Sensor”) was cut short to fit precisely at the carotid arteries and then secured in the bottom of the animal bed before being carefully placed around the throat of the mice. When all three mice were mounted on the animal beds and placed in the habituation box, the MRI sequence sounds were played, and physiological and visual recordings were commenced. After each habituation session, all *fecal boli* for each mouse were collected and dried overnight before weighing. Physiological data recordings were done with a sampling rate of 1 Hz and preprocessed using an in-house MATLAB script while statistical analyses were done in Prism 9 software.

#### Fecal Corticosterone Metabolite Assessment

Fecal corticosterone metabolite (FCM) levels were assessed at multiple timepoints during the experiment using the subset of animals (*n* = 18). The timepoints for fecal sample collection were (1) after 2 weeks of stable acclimatization and prior to any animal handling (pre handling) (2) 24 h after every third day of MRI habituation (3) 14 days after end of MRI habituation (post training) (Figure 1). We use FCM assessment to non-invasively obtain a direct measurement of the animal’s stress levels during our awake MRI habituation protocol.

Fecal samples were collected by carefully placing the mice into single cages with clean bedding material mixed with a handful of bedding material from their home cage. This was done to minimize the novelty of the environment in the new cages. After a period of approximately 1 h the animals were gently transported back to their home cage. All the excreted fecal samples from that period were collected with forceps and stored at –20°C. At the end of the experimental period all fecal samples were prepared in duplicates and in accordance with the manufacturer’s instructions^2^ FCM concentration levels were analyzed using a chemiluminescent immunoassay ELISA kit as described in the manufacturer’s instructions (Arbor Assays, Ann Arbor, MI, United States). The chemiluminescence was read with a microplate reader (Synergy HTX Multi-Mode Reader, BioTek, Santa Clara, CA, Unites States) and concentrations were determined in pg/mL but later converted to ng/mg. Statistical analysis was conducted using Prism 9 software.

### Automated Light/Dark Test Apparatus

Behavioral data were collected using the light/dark anxiety transition test (LDT) to examine anxiety-like behavior in young adult mice. Four individual sessions of LDT were conducted for each mouse: After the surgical recovery period ended (LD1)—baseline. One day after the first habituation session (LD2), 1 day after the last habituation session (LD3), and 2 weeks after the last habituation session (LD4) (Figure 1A). The automated setup consists of an IR-translucent light/dark box (Noldus, MMDLx-I001_V30) and an IR camera (Basler acA1300-60gmNIR, Basler) mounted in the ceiling alongside two indirect IR light sources (Lux). The box measured 20 × 60 cm in total (Light compartment: 40 × 20 × 20 cm, Dark compartment 20 × 20 × 20 cm). A small doorway in the middle connects the two compartments. Before each session, mice were acclimatized to the room for a minimum of 30 min. Mice were then placed in the center of the light compartment, and recordings were automatically initiated after 2 s of tracking validation done by the software. Mice were allowed to freely move and explore the two compartments for 10 min. Between sessions, the box was carefully cleaned with 70% ethanol and airdried for 5 min to ensure the odors of the previous animal had been removed. The number of transitions between the compartments, the total movement, and total time spent in the light compartment were extracted from EthoVisionXT software (Noldus), preprocessed using MATLAB-R2019a, while Prism 9 Software was used for statistical analysis.

### Statistical Analysis

All data are presented as mean ± standard error of the mean (SEM) unless specified otherwise. For statistical analysis of single groups (males: females) we conducted a one-factor ANOVA using a *post hoc* Dunnett’s test for multiple comparison with Day 1 as control for both males and females unless specified otherwise. Dunnett’s test was selected because we used Day 1 as control and were not interested in comparisons with other days. Results were considered statistically significant at *P*< 0.05. For statistical analysis of both groups (males vs. females) we conducted a two-factor mixed ANOVA using Holm-Sidak’s test for multiple comparison unless specified otherwise. Here, Holm-Sidak’s test was selected as it is more powerful than the Bonferroni test, and we did not need to compute the confidence intervals. Results were considered statistically significant at *P* < 0.05.

## Results

### Assessment of Animal Discomfort During Habituation

**FIGURE 3:**
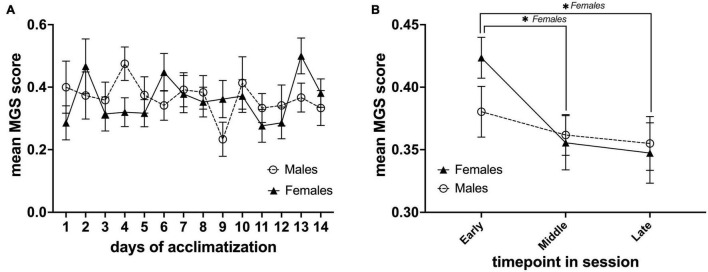
Daily mean MGS score during the awake MRI habituation period and within each MRI habituation session for male (*n* = 8) and female (*n* = 7) mice. **(A)** Mean MGS score for each sex of the different days. **(B)** Mean MGS score for each sex at the early, middle, and late time points within all sessions (14 days). Data are presented as mean ± SEM.

Our IR video recording setup allows us to observe the behavior and capture the facial expressions of the animals during each MRI acclimatization session (Figure 2F). From our observations, it is clear that the animals were moving the lower part of their body (this part was not fixated) when initially restrained but remained rather calm for the remainder of the session. In order to assess mouse discomfort during the awake MRI habituation sessions, we estimated their discomfort level at three different time points for each session using the MGS. Images from six mice were omitted from analysis due to out-of-focus images. Mice exhibited low discomfort levels during the awake MRI habituation period (mean value for all days; males = 0.36 ± 0.014 SE, *n* = *8*, females 0.36 ± 0.018 SE, *n* = *7*) (Figure 3A). For day-to-day comparison, the MGS score for all timepoints for each session was averaged for each animal, before averaging across animals for both the male and female group. For both males and females, no difference was found in the daily mean MGS score fluctuations (males: *P*> 0.05; females = *P*> 0.05 in a Dunnett’s multiple *t*-test with Day 1 as control) (Figure 3A). A two-factor mixed ANOVA did not reveal any differences when comparing males vs. females [*F*_(13,182)_ = 1.185, *P*> 0.05, main effect of days in a two-factor mixed ANOVA of mean MGS score × days] (Figure 3A). To investigate whether the mice exhibited different discomfort levels during a single session, we compared the mean MGS score between three different time points (early, mid, and late) within each session for males and females. We observed a continuous decrease in the mean MGS score across the timepoints for males and females. No difference was found when comparing males vs. females in a two-factor mixed ANOVA main effect (*P*> 0.05) (Figure 3B). However, we found that the mean MGS score for females decreased significantly from the early time point to the middle and late time points [*F*_(2,39)_ = 3.672, *P*< 0.05, row effect of time point in session using a two-factor mixed ANOVA of mean MGS score × time point in the session, followed by multiple comparisons of row effects of time point in the session using Holm-Sidak’s test; *P< 0.05 vs. mean MGS score] (Figure 3B).

### Heart Rate Recordings

**FIGURE 4:**
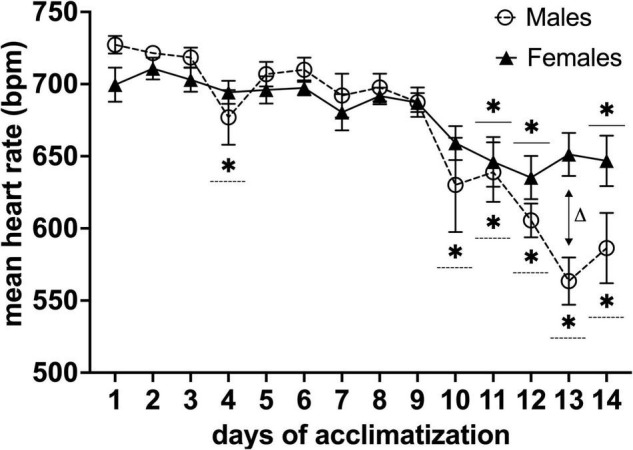
Effects of awake MRI habituation on heart rate for male (*n* = 11) and female (*n* = 13) mice. *P< 0.05 in a Dunnett’s test (day 1 as control). Δ*P* < 0.05 in Holm-Sidak’s multiple comparison using two-factor mixed ANOVA with sex × days of acclimatization. Data are presented as mean ± SEM.

Mean heart rates during the acclimatization period decreased significantly on the fourth day and from the tenth day onward of habituation for males (*n* = 11) and the 11, 12, and 14th day for females (*n* = 13) (*P < 0.05 in a Dunnett’s test, with day one as control) (Figure 4). Moreover, we found a difference in mean heart rate for males vs. females [*F*_(13,283)_ = 2.784, *P*< 0.001, main effect of days in a two-factor mixed ANOVA of acclimatization × days]. Multiple comparisons of the main effects of days using Holm-Sidak’s test vs. mean heart rate between males and females on each day, revealed a difference on day 13 of habituation (Δ*P*< 0.001) (Figure 4).

### Body Weight Fluctuations Throughout the Experimental Period and Weight of *Fecal boli* During Habituation Period

**FIGURE 5:**
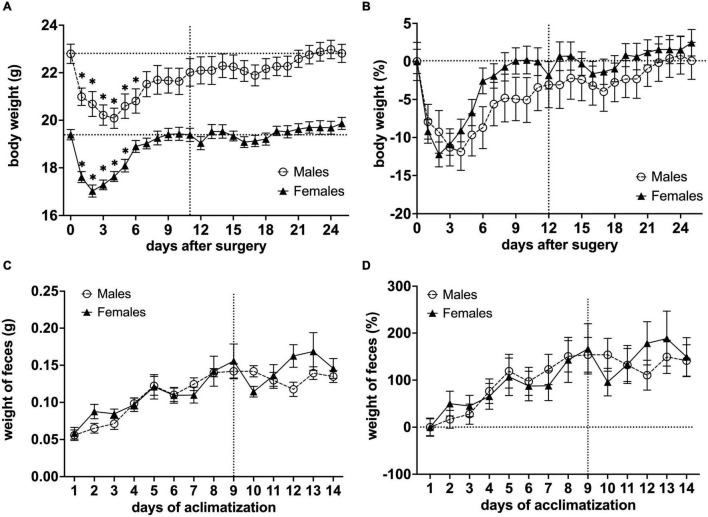
Effects of surgical procedures and awake MRI habituation on mouse body weight fluctuations and weight of *fecal boli* throughout the awake MRI habituation. **(A)** Mean body weight of males and females (*P< 0.05 vs. body weight after surgery with day 1 as a control in a Dunnett’s test). **(B)** Relative change in mean body weight with Day 0 used for baseline correction. Note that the vertical line on **(A,B)** indicates the start of awake MRI acclimatization. The horizontal lines on **(A)** indicate the mean body weight post-surgery for males and females, respectively, while the horizontal line on **(B)** indicates the corrected baseline (0%). **(C)** Mean weight of *fecal boli* during habituation period. **(D)** Relative change in mean weight of *fecal boli* with Day 0 used for baseline correction. Note that the vertical lines on **(C,D)** indicate when awake MRI habituation length reached the maximum (180 min), while the horizontal line on **(D)** indicates corrected baseline (0%).

The total weight of a head-holder, ceramic rod, and dental-cement was 0.89 g ± 0.08 SEM (*n* = 36*).* This weight was subtracted from the mouse weight post-surgery. We observed post-surgery weight loss in all animals. For males, body weight decreased for 6 days after the surgical implantation of the head-holder (*P< 0.05 vs. body weight after surgery with day 1 as control in a Dunnett’s test), and it took 22 days before they regained their initial weight. Meanwhile, the body weight of females decreased for 5 days (*P< 0.05 vs. body weight after surgery with day 1 as control in a Dunnett’s test), and initial weight was reached 9 days post-surgery (Figure 5A). In order to compare body weight between sexes, relative change to baseline (Day 0) was calculated (Figure 5B). We found that the distinctive time course of the body weight for males and females was significantly different [*F*_(1,572)_ = 14.48, *P*< 0.001, column effect using a two-factor mixed ANOVA of sex × days]. However, multiple comparisons using Holm-Sidak’s method did not reveal any days of significance. For the weight of *fecal boli* samples, we observed a continuous increase for both males and females as the length of the awake MRI habituation increased, but not after the habituation length reached the maximum (180 min) (Figures 5C,D).

### Light-Dark Test

#### Total Distance Traveled

**FIGURE 6:**
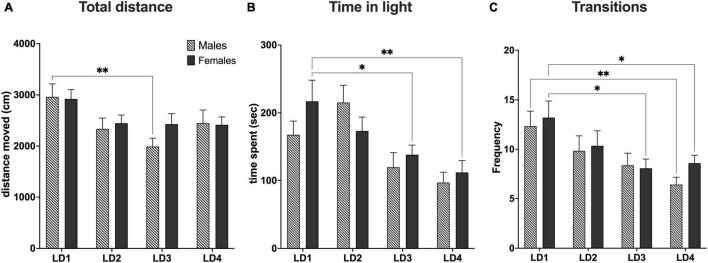
Behavioral data from light-dark test. **(A)** Total distance traveled during the light-dark test (10 min). **(B)** Total time spent in the light compartment **(C)**. Total amount of transitions between the light and dark compartment. LD1 (light-dark test performed before surgery; males, *n* = 15; females, *n* = 15). LD2 (light-dark test 1 day after first habituation day; males, *n* = 19; females, *n* = 20). LD3 (light-dark test performed after last habituation day; males, *n* = 17; females, *n* = 19). LD4 (light-dark test performed 14 days after habituation; males, *n* = 17; females, *n* = 17). *P< 0.05; ^**^P< 0.01. Data are presented as mean ± SEM.

Overall, we observed that both groups exhibited decreased locomotor activity from LD1 through LD2 and LD3 with males exhibiting the biggest decrease. In LD4, we were able to observe a slight increase in locomotor activity from LD3 for both groups. However, statistical analysis for total distance traveled (DIS) did not reveal any sex difference [*F*_(3,131)_ = 0.6230, *P*> 0.05, main effect using two-factor mixed ANOVA sex × LD]. However, a DIS × LD difference was observed [*F*_(3,131)_ = 4.481, *P*< 0.01, row effect using two-factor mixed ANOVA of distance × LD]. *Post hoc* multiple comparison using Dunnett’s test revealed statistical significance in the LD1 vs. LD3 comparison for males (^**^P< 0.01) indicating that mice displayed decreased locomotor activity in LD3 when compared to baseline (Figure 6A).

#### Time in Light Compartment

In general, we observed that female mice spent less time in the light compartment as the light dark test progressed from LD1 through LD4. For male mice we observed that the time in the light compartment increased from LD1 to LD2 whereafter time spent in light decreased from LD2 through LD4. Statistical analysis of time spent in the light compartment (TSL) did not reveal any sex difference [*F*_(3,131)_ = 1.661, *P*> 0.05], main effect using two-factor mixed ANOVA of sex vs. LD). A LD effect in TSL [*F*_(3,131)_ = 9.176, *P*< 0.0001 row effect using a two-factor mixed ANOVA] followed by Dunnett’s test revealed a difference in LD1 vs. LD3 (*P< 0.05) and LD1 vs. LD4 for females (^**^P< 0.01) (Figure 6B).

#### Transitions Between Light and Dark Compartment

For transitions (TNS), we observed that male mice had fewer transitions than female mice across all four LD tests except LD3, however no sex difference were found [*F*_(3,131)_ = 0.3166, *P*> 0.05] main effect using two-factor mixed ANOVA of sex × LD). However, significant was seen for TNS × LD [*F*_(3,131)_ = 6.111, *P*< 0.001, using a two-factor mixed ANOVA]. *Post-hoc* multiple comparison using Dunnett’s test revealed statistical significance in the LD1 vs. LD3 (*P< 0.05) and LD1 vs. LD4 (*P< 0.05) for females and LD1 vs. LD4 (^**^P< 0.01) for males (Figure 6C).

### Assessment of Fecal Corticosterone Metabolites Levels

**FIGURE 7:**
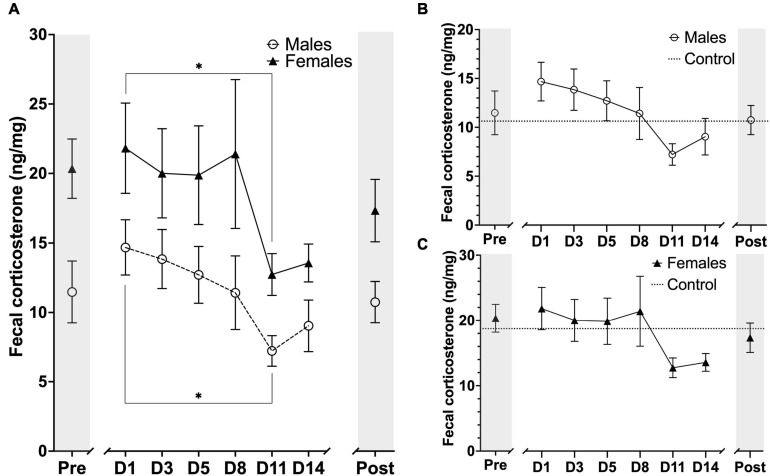
Timeline representation of fecal corticosterone metabolite (FCM) concentration levels at the sampling timepoints during the experimental period. Units are displayed in FCM ng/mg of fecal matter. **(A)** FCM concentration for males (*n* = *6*) and females (*n* = *6*) at each sampling timepoint. **(B)** FCM concentration for males (*n* = *6*) and male controls (*n* = 3). **(C)** FCM concentration for females (*n* = *6*) and mean FCM concentration for female controls (*n* = 3). The markers on x-axis indicates the different sampling timepoints (Pre = after stable acclimatization; D1–14 = during the 14 days of MRI habituation; Post = 14 days after last MRI habituation day). The horizontal lines for control on figure B, C indicate mean FCM concentrations for the whole experimental period. Data points are presented as mean ± SEM. *P < 0.05.

Concentration of fecal corticosterone metabolites were assessed throughout the experiment at eight different sampling timepoints, with one sample point prior to animal handling, six sample points evenly spread-out during the length of the awake MRI habituation and one sample point 14 days after the MRI habituation had ended. As expected, we observed that the mean FCM concentration for females was generally higher than for males throughout all of the sample points in the experiment. For both groups we observed that day 1 of MRI habituation (D1) induced an increased response in mean FCM concentration when compared to the mean concentration at pre handling level. For male mice the mean FCM concentrations decreased continuously until D11 whereafter we observed a slight increase for the D14 and Post timepoints. A similar trend could be observed for the females except D8 showed a brief increase in mean FCM concentration (Figure 7A). Statistical analysis did not reveal any sex differences [*F*_(7,79)_ = 0.2224, *P*> 0.05] main effect using two-factor mixed ANOVA of sex × habituation). To compare the effect of MRI habituation on FCM concentrations for each group, we used the D1 mean FCM concentration as reference points in six-paired two-sample two-sided *t*-tests. A significant decrease in mean FCM concentration from the first day (D1) of MRI habituation to the eleventh day (D11) FCM [*t*_(5)_ ≥ 2.998, *P*≤ 0.0302] for both male and females mice (Figure 7A) coinciding with the first time point where animal heart rate is significantly lowered and indicative that FCM concentrations decreased with continuous awake MRI habituation. No significant differences were found in either group on other days when using D1 mean FCM concentration as reference point.

We also compared the animals in each group (*n* = 6) to a control group of their own sex (*n* = 3) (Figures 7B,C). In general, we observed very large fluctuations of mean FCM concentration levels in the control groups for both males and females during the awake MRI habituation and statistical analysis did not reveal any difference between male vs. control nor female vs. control.

## Discussion

### Awake Mouse Magnetic Resonance Imaging Habituation Protocol

Our awake MRI habituation protocol were designed to include as many of the current best practices in order to present a protocol optimized for animal welfare and reduction of animal stress. For example, we implemented a progressive awake MRI habituation protocol (Figure 1B) in similar fashion to previous reports using rats and mice (Stenroos et al., 2018; Han et al., 2019; Chen et al., 2020; Cover et al., 2021) to better account for the potential short and long-term pain and stress that non-progressive protocols might exert on the animals (Stenroos et al., 2018). In addition, we provided earplugs to the animals to help minimize the stress and potential ear damage induced by the loud acoustic MRI noises. Our head-fixation protocol also allowed animals free movement of the lower body. It has previously been suggested that researchers should include behavioral outcome measurements in addition to stress-related physiological measurements, as the normal range of physiological parameters alone does not fully explain the long-term effects of awake MRI habituation on mice (Low et al., 2016). Therefore, we implemented the light-dark test to measure anxiety-like and locomotor behaviors in addition to recordings of heart rate and FCM measurements. Lastly, our setup did not require sedation for head-fixation of the animals, which is consistent with previous reports (Yoshida et al., 2016; Han et al., 2019; Chen et al., 2020) and does away with the concern for the effect of lingering anesthetics. Our habituation protocol relies on the similarity between the scan simulation environment and the actual scan session. Previous studies have suggested including actual imaging sessions in the habituation protocol as beneficial for reducing animal stress (Chen et al., 2020) and to reduce the total amount of animal movement (Dinh et al., 2021). For most labs, however, scanner resources are too scarce to include an actual MRI scanner in the habituation so we opted for a habituation protocol using a simulated scanner environment only to ensure our study is relevant to as many labs as possible. For future work it may be beneficial to include resting days as implemented in similar studies (Russo et al., 2021). However, our FCM data shows that if habituation is not maintained stress of handling increases again (Post time point in Figure 7A, both sexes). This must be taken into account when incorporating rest days into an experiment timeline.

### Effects of Surgical Procedures and Awake Magnetic Resonance Imaging Habituation on Heart Rate, Body-Weight, and Weight of *Fecal boli* in Male and Female Mice

We evaluated the time courses of several stress indicative physiological, behavioral, and biomarker parameters (heart rate, body weight, weight of *fecal boli*, and fecal corticosterone levels). For heart rate measurements, we observed that both groups elicited decreased mean heart rates after continuous awake MRI habituation (10 days for males; 11 days for females) (Figure 4), which was longer than anticipated as previous studies report a decrease in mean heart rate after only 7 days (Yoshida et al., 2016). Furthermore, we observed that males displayed lower mean heart rates ranges on the last 3 days of the awake habituation protocol than females. When we compared the distinct time course of mean heart rate for males and females, we detected a significant difference (Figure 4), suggesting that awake MRI habituation impose more stress on female than male mice, which is in concordance with reports that female rodents are more sensitive to physiological and physical stressors (Rincón-Cortés et al., 2019). Finally, it has to be noted that our MouseOx pulse oximetry system was indeed capable of providing other measurements such as arterial saturation and breath rate, which might have been valuable to include. However, due to the high sensitivity of the sensors to small movement, these measurements were too noisy to include.

The body weight of both groups decreased rapidly the days after surgical implantation of the head-holder. This effect was expected based on other studies that utilized surgical implants for head-fixation (Yoshida et al., 2016; Madularu et al., 2017; Stenroos et al., 2018). We observed similar daily fluctuations of mean body weights of males and females; however, some distinct differences were present (Figures 5A,B). Although both males and females recovered significant body weight after only five (females) and six (males) days, it took much longer for males to regain their initial body weight loss post-surgery. However, none of the mice exceeded 20% of body weight loss post-surgery, and all were able to regain their body weight, indicative of a successful postoperative recovery. Furthermore, we observed a steady weight gain throughout the experimental period for both groups, indicating that mice eating behavior was not affected. However, in future studies, recovery may be accelerated by administering isotonic glucose, or other sugar-based liquids, in the days following surgery. Excessive excretion has been established as indicative of stress and feces weight used as a measure of it (Miyata, 1992). In our study, the mean weight of *fecal boli* in both groups increased as the habituation length increased but stagnated after the habituation length reached 180 min (Figure 5C). This indicates that prolonged MRI habituation does induce increased excretion in the animals. However, the mean *boli* weight during the habituation period using 180 min sessions was in the range of those found in a similar study (Yoshida et al., 2016), indicating that the excretion was not excessive. Taken together, these findings indicate that the surgery and habituation protocol does not affect the animals’ basic wellbeing (recovery, feeding, weight gain, and weight stability) of the animals.

### Effects of Awake Magnetic Resonance Imaging Habituation on Mouse Grimace Scale and Light-Dark Test Outcomes in Male and Female Mice

While the utmost care is taken to minimize animal discomfort during the entire habituation procedure, it is unrealistic to avoid discomfort and stress completely. For this reason, we monitored animal discomfort and anxiety response using the MGS and LDT. The average MGS score for both groups was extremely low (< 0.36) throughout the experiment, indicating that mice only experience mild discomfort during habituation. We did however observe that female mice displayed slightly increased discomfort levels during the initial minutes just after being restrained (Figure 3B). This suggests that male mice exhibit greater stress/pain resilience than females. In a few cases, for the longer (≥120 min) awake MRI sessions, we observed a layer of eye secretion that had developed around the eyes of the mice. In our experience, this typically occurs when mice are highly aroused for longer periods of time, indicating that some mice were very alert during the long sessions. However, more research is needed to fully explain this phenomenon. For example, it would be interesting to correlate eye secretion occurrences with EEG data.

The light-dark test (LDT) was used to assess anxiety-like and locomotor activity behavior during MRI habituation. The LDT test utilizes the inherent behavior of mice to avoid bright open spaces (light compartment) and preference for a safe environment (dark compartment). Increases in anxiety-like behaviors and decreases in locomotor activity are adaptive sickness behaviors in that they minimize risk taking. It was previously shown that prolonged restraining, similar to those methods applied in awake MRI habituation, can cause short-term increases in the stress hormone corticosterone and long-term alterations in physiological responses to painful stimuli (Low et al., 2016). In addition, it was shown that chronic stress can induce changes in anxiety and modify locomotor activity levels (Naert et al., 2011). In general, we observed that mice spent more time in the dark compartment than the light compartment consistent with the light-dark test as a general measure for anxiety-like behavior (Banasikowski et al., 2015). Both groups exhibited similar locomotor activity levels in all LDT’s except on LD3 where we detected a decrease in locomotor-activity for males. After the 14-day break, on LD4, we could not detect this effect anymore (Figure 6A). This suggests that male mice might be more prone to decrease their locomotor activity when exposed to stressful environments than females. Last, we assessed time spent in the light compartment and total transitions, which are typical measurements of anxiety-like responses. Although, statistical analysis did not detect any difference between males and females on time in light, we did observe some individual differences. Females spent less time in the light compartment on LD3 which is indicative of an increased anxiety-like behavior. This increase remained even 2 weeks after the MRI habituation had ended on LD4 (Figure 6B). For frequency of transitions in the LDT’s we found no difference between males and females, but did however detect individual differences over the time course of the conducted LDT’s. For females we detected a decrease in transitions on LD3 indicating an increase in anxiety-like behavior after 14 days of MRI habituation. This effect remained detectable for the females 14 days after the MRI habituation ended (LD4) indicating that our awake MRI habituation is capable of inducing a long-term anxiety effect. We also detected a significant decrease in transitions for males on LD4 but not on LD3. It is unclear what the reason for this observation would be. In general, based on our data from the facial video recordings and our behavioral data from the LDT, we found that while overall animal anxiety and discomfort is very low female mice do display higher discomfort and show more anxiety-like behaviors than males, when exposed to our 14-day awake MRI habituation protocol.

### Discomfort, Stress, and Anxiety in Male and Female Mice

Awake animal MRI is valuable because it avoids the physiological perturbations caused by anesthesia. However, stress and anxiety are known to alter brain chemistry, physiology, and behavior (Luine et al., 2001; Vestergaard-Poulsen et al., 2011; Khan et al., 2016, 2018, 2019). For example in studies of chronic stress using rodent restraint models, body weight has been shown to decrease over time (Woo et al., 2018) and is accompanied by an increase in the plasma level of the stress hormone corticosterone (Kim et al., 2013). Our awake MRI habituation protocol was developed to also allow for MRI studies of extended duration where sessions need to last longer than 2 h. We therefore implemented a rather long MRI habituation protocol of 14 days including awake MRI habituation sessions lasting up to 3 h (Figures 1A,B). Other MRI habituation protocols currently found in the literature, are typically employing awake MRI habituation sessions only lasting ≤ 2 h (Bergmann et al., 2016; Yoshida et al., 2016; Madularu et al., 2017; Desjardins et al., 2019; Tsurugizawa et al., 2020). Therefore, our MRI habituation protocol might be considered more stressful. However, our data shows that habituation for extended scan sessions is possible as mean heart rate values in male and female mice were comparable to normal heart rates found in unperturbed conscious mice after 10 (male) and 11 (female) days of MRI habituation (Kass et al., 1998). In addition, we found that mean FCM levels from the first day of MRI habituation were significantly decreased on the 11th day of MRI habituation for both male and female mice (Figure 7A) coinciding with the time at which heart rate has normalized (Figure 4). Furthermore, mean FCM concentrations for both male and female on the last day of MRI habituation appear below that of pre handling levels (Figure 7A). This is consistent with other groups who reported decreased FCM concentration levels after several days of awake MRI training (King et al., 2005; Tsurugizawa et al., 2020; Russo et al., 2021). Lastly, during our MRI habituation period, both male and female mice were able to recover, maintain and increase their body weight. On the basis of our available results across the different stress-indicative parameters, it is therefore evident that mice are able to habituate to the stressful MRI environment, even during application of an extended MRI habituation protocol. However, while both sexes are successfully habituated in our study, our wide characterization does reveal lingering differences in anxiety related behavior exist post-training (Figure 6) and in corticosterone levels (Figure 7). These differences are expected based on the existing literature on stress response in male and female mice. Likely, these differences will not affect MRI derived parameters or at least their effect will be much less than the effect of anesthesia. However, these details have implications for studies where both awake MRI and behavior tests will be used to characterize an animal model. Firstly, both sexes should be included as inherent physiological differences (e.g., stress hormone levels) exist even after habituation. Secondly, separate groups should be used for awake MRI and behavior analysis as otherwise the behavior results may be influenced by sex-specific, lingering effects of the MRI habituation. These recommendations apply to both awake fMRI studies and quantitative MRI studies for which our protocol is aimed.

## Conclusion

This study is the first to examine the effects of surgical and restraint procedures associated with awake MRI habituation on stress-related physiological, biological and behavioral parameters in both male and female mice. We find that while sex differences exist, male and female mice can be successfully habituated for extended awake MRI scan sessions. Using awake mice for MRI research purposes requires very time-consuming habituation compared to experiments using anesthetized animals. However, many aspects of brain physiology cannot be properly investigated under anesthesia, and awake animal MRI methods are therefore crucial for modern neuroscience.

## Data Availability Statement

The raw data supporting the conclusions of this article will be made available by the authors, without undue reservation.

## Ethics Statement

The animal study was reviewed and approved by the Danish Animal Experimentation Council.

## Author Contributions

BH and TL designed the study, analyzed the data and interpreted results. BH acquired funding. TL built the training hardware, performed the surgeries, animal training and data acquisition, and wrote the initial manuscript draft. All authors edited the manuscript and contributed to the experimental setup.

## Conflict of Interest

The authors declare that the research was conducted in the absence of any commercial or financial relationships that could be construed as a potential conflict of interest.

## Publisher’s Note

All claims expressed in this article are solely those of the authors and do not necessarily represent those of their affiliated organizations, or those of the publisher, the editors and the reviewers. Any product that may be evaluated in this article, or claim that may be made by its manufacturer, is not guaranteed or endorsed by the publisher.

## Footnotes

1
https://github.com/porridgeJoe

2
https://www.arborassays.com/assets/steroid-solid-extraction-protocol.pdf
